# Baduanjin Qigong Intervention by Telerehabilitation (TeleParkinson): A Proof-of-Concept Study in Parkinson’s Disease

**DOI:** 10.3390/ijerph18136990

**Published:** 2021-06-30

**Authors:** Livia P. Carvalho, Simon Décary, Isabelle Beaulieu-Boire, Rosalie Dostie, Isabelle Lalonde, Émilie Texier, Laurence Laprise, Elizabeth Pepin, Mélodie Gilbert, Hélène Corriveau, Michel Tousignant

**Affiliations:** 1 Faculty of Medicine and Health Sciences, School of Rehabilitation, Université de Sherbrooke and Research Centre on Aging, Sherbrooke, QC J1H 5N4, Canada; livia.pinheiro.carvalho@usherbrooke.ca (L.P.C.); simon.decary@usherbrooke.ca (S.D.); rosalie.dostie@usherbrooke.ca (R.D.); isabelle_lalonde.cisssmo16@ssss.gouv.qc.ca (I.L.); emilie.texier@usherbrooke.ca (É.T.); laurence.laprise@usherbrooke.ca (L.L.); elizabeth.pepin@usherbrooke.ca (E.P.); melodie.gilbert@usherbrooke.ca (M.G.); helene.corriveau@usherbrooke.ca (H.C.); 2 Centre Intégré Universitaire en Santé et Services Sociaux de l’Estrie-Centre Hospitalier Universitaire de Sherbrooke (CIUSSS-de-l’Estrie-CHUS), Sherbrooke, QC J1J 3H5, Canada; isabelle.beaulieu-boire@USherbrooke.ca

**Keywords:** physical therapy, exercise, rehabilitation, parkinsonism, telemedicine

## Abstract

Many people living with Parkinson’s Disease (PD) face issues with healthcare services, including delays in diagnosis and treatment, as well as limited access to specialized care, including rehabilitation programs. Non-motor and motor signs and symptoms typically observed in people with PD, such as tremor, rigidity, postural instability, bradykinesia, and freezing are particularly disabling and have been associated with falls, fractures, hospitalizations, and a worse quality of life. Baduanjin Qigong (BDJ) programs have been proven potentially effective in improving physical outcomes and reducing the incidence of falls in PD. The aim of this case report, proof-of-concept, study was to explore the adherence, feasibility, acceptability, and potential efficacy of a BDJ program offered via telerehabilitation in people with PD living in the community. Two participants performed semi-supervised exercise sessions at home, twice a week (over eight weeks) using the TeraPlus platform. Adherence, adverse events, and feasibility (technical implementability), acceptability (patient satisfaction), patient-reported, self-reported, and performance outcomes were measured. Results were based on single-subject descriptive data, minimal detectable change, and anchor-based minimally important difference. Our findings suggest that the intervention seems feasible with no major technical issues or adverse events, and high adherence; acceptable (patient satisfaction); and potentially effective to improve markers of walking performance (gait speed, balance), and quality of life (activities of daily living, mobility).

## 1. Introduction

The dopaminergic deficit in patients with Parkinson’s Disease (PD) is responsible for the occurrence of several motor and non-motor signs and symptoms typically observed in people with PD [1]. Among the motor symptoms, resting tremor, rigidity, postural instability, slowness of movement, and freezing while walking are particularly disabling [2] and, because pharmacological treatments cannot fully compensate or relieve them, greater issues arise. Recurrent falls (~39%, ~20 falls per year) [3]; frequent rehospitalizations to acute care (patients with a history of hospitalization are 1.7 times more likely to undergo re-hospitalization compared to those without a prior event) [4]; and admission to long-term care facilities (5–10% prevalence of PD among all patients) [5], depression, and cognitive impairment, among others, have been reported to significantly impact the quality of life of patients with PD [6] and, ultimately, to increase their likelihood of developing other comorbidities and dying prematurely [7].

In Canada, the prevalence of parkinsonism, including PD, the most common and fastest growing neurodegenerative disorder worldwide [7], is estimated to double by 2031 and the number of new cases to increase by more than 50% over this period [8]. In an aging population, it is even more worrisome that the burden of this disease increases significantly with age, with those aged 65 and older (~20% of the population) accounting for about 85% of cases [8,9]. It is therefore not surprising that the direct or indirect costs associated with PD are estimated at approximately $1.2 billion per year, with morbidity costs representing the larger component of the indirect costs for PD [10,11].

It becomes therefore imperative to identify strategies to prevent further acute events and subsequent medical interventions as well to improve, or at least maintain, skills and capacities that enable patients with PD to better engage in their daily activities and move around in the community more often and more effectively.

However, many people living with PD face issues with delays in diagnosis and treatments as well as limited access to healthcare services that are proven to help in coping and managing the difficulties inherent to the disease [12,13]. A recent Canadian survey [13] showed that around 1 in 3 people with PD reported their access to movement disorder clinics and specialists to be poor or very poor, and only half of the diagnosed patients reported to have been a patient in such clinics. When asked about the services that would help patients to live better with PD, both patients and caregivers rated physical activity programs (73%) and physical therapy (52%) most highly.

Telemedicine strategies in neurological patients, more specifically telerehabilitation [14,15,16,17,18], seems more than ever a promising avenue and an inevitable consequence of the huge advances made in the past decades in terms of the development of technology-based approaches and equipment that can be easily incorporated into clinical practice and adapted to multiple health conditions and rehabilitation settings. This strategy fills a healthcare need to care for a greater number of patients simultaneously in an already overloaded health system while expanding access to rehabilitation programs. Telerehabilitation has the potential to be used as a hybrid approach to in-person rehabilitation, to provide non-emergent care and/or as a way to provide follow-up support to low-risk patients, or to better manage discharge processes [19], especially for those living in remote areas or areas where there is no such healthcare service [20].

Finally, this modality of healthcare provided can help mitigate the eventual impossibility of healthcare professionals to provide in-person care in challenging contexts, such as the one all healthcare systems worldwide have been dealing with during the most recent pandemic [21,22], without interrupting ongoing treatments or increasing patients and practitioners’ exposure to unnecessary harm.

A variety of physical interventions in PD [23,24,25], including Baduanjin Qigong (BDJ) [26,27,28,29] and other similar interventions, such as Tai Chi training programs [30,31,32,33,34,35,36,37], have been proven potentially effective in improving gait parameters, balance, physical function, walking capacity, mobility, physical activity participation, and reducing the incidence of falls in patients with PD. In the context of telerehabilitation, despite some lack of outcome standardization and mid- to long-term follow-up studies, recent evidence suggests that these types of technology-based interventions and delivery methods are feasible, safe, and more or as effective and costly as conventional in-person approaches in improving motor components associated with PD [17,38,39]. However, to the best of our knowledge, the combination of a mind-body type of exercise program delivered via telerehabilitation has not been tested in this population and more research is needed. Similar telerehabilitation programs using the platform proposed in this study have led to promising results in older adults after discharge from an acute rehabilitation setting [19] and patients with other health conditions, such as post-stroke patients returning home without an intensive rehabilitation program [40] and post-knee arthroplasty [41]. The platform has several features that make it easy for use by therapists: it has a flexible design that can be adapted to different types of rehabilitation programs and enable the installation of several cameras in multiple spots; it can integrate sensors that provide real-time physiological data; and it provides the possibility of applying online questionnaires whenever needed. In this context, the aim of this proof-of-concept study was to explore the adherence, feasibility, acceptability, and potential efficacy of a BDJ exercise program offered via telerehabilitation in people with diagnosed PD. Specifically, the objectives were: (1) to investigate whether this type of telehealth intervention seems feasible and acceptable from two main points of view: technical implementation/adherence and patient satisfaction with the service provided; and (2) to explore the potential extent of improvement in aerobic capacity, balance, and health-related quality of life in response to this modality of intervention.

## 2. Material and Methods

### 2.1. Study Design, Sample Characteristics, and Ethics

This was a case report, pre–post, proof-of-concept study, carried out over a period of 8 weeks. Two persons with early- to mid-stage PD for at least three years—defined according to the Movement Disorders Society’s diagnostic criteria [42]—were identified by a neurologist specializing in PD from the CIUSSS de l’Estrie-CHUS (Sherbrooke, QC, Canada). Eligibility criteria included the presence of postural instability measured by the “pull test” (Movement Disorders Society—sponsored revision scale of the Unified Parkinson Disease Rating scale, MDS-UPDRS) [42]; home access to the internet; and, for the purpose of safety, presence of a caregiver at home. Patients with a suspected cognitive deficit (Mini-Mental State Examination, MMSE <24) [43] were not considered eligible for study enrollment. Included participants were optimally medicated (levodopa or carbidopa, with or without other dopaminergic therapies) throughout the study duration.

The study was conducted according to the guidelines of the Declaration of Helsinki and approved by the Institutional Review Board of the CIUSSS de l’Estrie-CHUS (#2020-3253, approved 2019-01-01). Written informed consent was obtained from participants involved in this study prior to enrolment.

### 2.2. Measures

Adherence to intervention (average sessions completed/total estimate sessions, number of supervised and unsupervised sessions, and reason for absence(s)); safety (adverse events, including falls during exercises), and technical issues (technical feasibility) experienced during the sessions were measured.

Acceptability was estimated through the questionnaire on participants’ satisfaction with the program provided (Telemedicine Satisfaction Questionnaire, TSQ), which included three factors: quality of care provided related to delivery of healthcare, accessibility, reliability, and attention (factor 1); similarity to face-to-face encounter (factor 2); and perception of the interaction related to communication through telemedicine (factor 3) [44].

**Figure 1:**
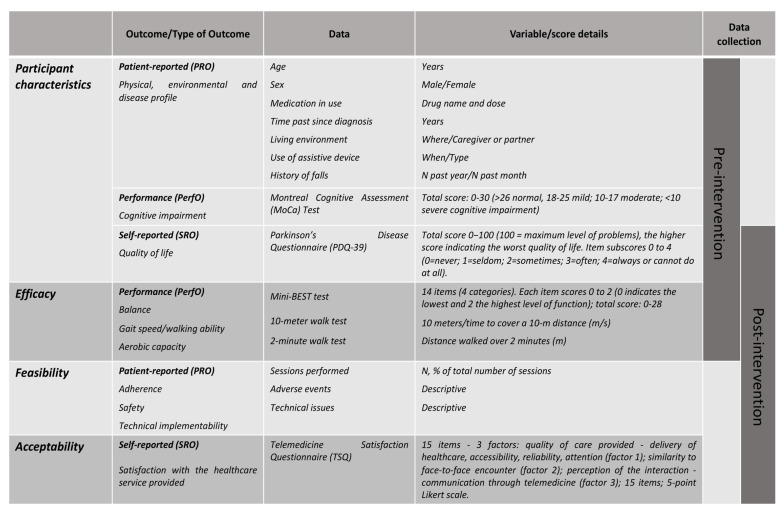
Detailed information about outcomes under study.

Patient-reported (PRO), self-reported (SRO), and performance (PerfO) outcomes [45], as detailed in Figure 1, were collected at the patient’s homes at two different timepoints: pre-training and one week after the end of the training program. Pre-training data collection included personal, environmental, and medical information, as well as the application of questionnaires and/or clinical tests for assessing quality of life (Parkinson’s Disease Questionnaire, PDQ-39) [46], cognitive impairment (Montreal Cognitive Assessment test, MoCA) [47], balance (Mini Balance Evaluation Systems Test, Mini-Best) [48], self-selected and fast-paced gait speed (10 m walk test) [49], and aerobic capacity (two-minute walk test) [50]. Post-training assessment included the balance, gait speed, and aerobic capacity tests, the PDQ-39 questionnaire, as well as the feasibility and acceptability outcomes.

### 2.3. Intervention: The Baduanjin Qigong Exercise Program

The BDJ training exercise program was delivered individually, at each patient’s home, through telerehabilitation, with direct remote supervision by a physiotherapist or a student in training in the 4th year of a professional Master’s degree. Each session consisted of a short preparatory phase (stretching exercises and abdominal breathing patterns) followed by a set of seven movements, adapted from the eight-section brocade BDJ that originally comprises eight movements, repeated a certain number of times. Due to major balance issues in people with PD, and for safety purposes due to nature of the intervention, one BDJ movement involving moving hands down the back and legs and touching the feet [48] was not included in our exercise program. Therefore, the program comprised seven sections, each set of movements being repeated four times. The movements are of low intensity, gentle, and slow, emphasizing static and dynamic postural control integrated with breathing patterns [51]. These were thoroughly explained and demonstrated to the participants prior to and throughout the entire session. A personalized approach was used in order to adapt the exercise movements to each patient’s ability and capacity to avoid compensations and injuries as a result of the prescribed program. The exercise program lasted eight weeks and included two supervised sessions and one unsupervised session, the latter aiming to develop the patients’ autonomy and motivation to later undertake exercises on their own without the close supervision of the therapist.

### 2.4. The Telerehabilitation Platform

**Figure 2:**
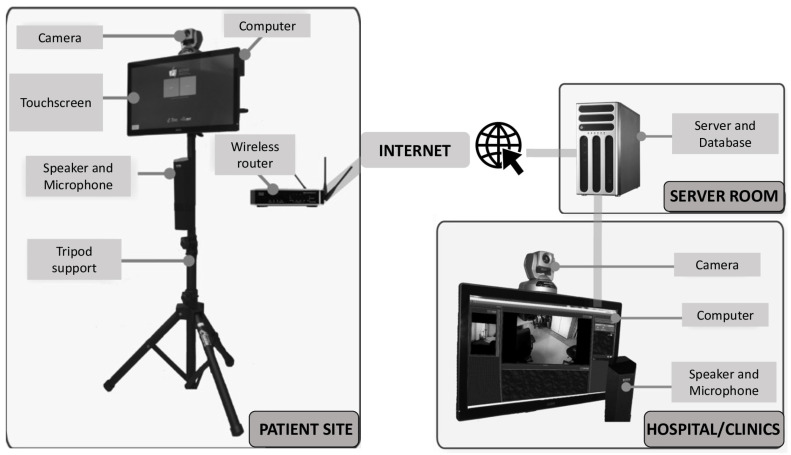
Representation of the telerehabilitation system used in the study.

The platform is based on a technological infrastructure previously developed by our team (TeraPlus software, ESTRAD, Sherbrooke, Canada). This infrastructure combines a clinical information system with videoconferencing components (h.264 network camera and microphone) and uses cameras controlled by the clinician from a software environment (Figure 2). This system provides a wide field of vision and a very high imaging quality in comparison to many other systems. The TeraPlus software, linked to the other components, ensures that the confidentiality of the patients’ medical information is respected. Compared to some others, our platform is intended to be easier for clinicians to use as they do not have to generate a link for each session, which is time-saving and convenient for both the clinician and the patient; it allows the clinician to take pictures during the session, which allows for the better planning and tracking of movement improvement over sessions; it shows a friendly visualization of the whole program schedule (past sessions and sessions to come); and, finally, it offers a user-friendly interface (e.g., access the session by clicking an ON/OFF icon) to enhance the patient’s ease-of-use perception. Prior to the initiation of the study, the necessary equipment was installed in the patients’ homes and instructions were provided for proper use.

### 2.5. Analysis

Since proof-of-concept studies are not powered to detect statistical differences, the results were based on the interpretation of single-subject descriptive data. Adherence, adverse events, feasibility, and acceptability outcomes were interpreted as descriptive. Pre–post meaningful changes for PerfOs were interpreted based on the minimal detectable change (MDC), which is a distribution-based approach to determine a minimal amount of change not due to random variation of measurement. For the pre–post PRO (PDQ-39), the interpretation of meaningful change was based on the anchor-based minimally important difference, which is the difference perceived to be meaningful to patients.

## 3. Results

### 3.1. Sample Characteristics

Table 1 presents the characteristics of the study participants, one woman (A) and one man (B) aged around 75 years old, living with the diagnosis of PD for 12 and 17 years, respectively. Despite the later diagnosis, participant A reported to have had more fall events in the past year; to need a walking aid to walk outside on her own; and demonstrated worse balance than participant B as per the Mini-Best test, more specifically the anticipatory (adjustments in preparation for expected movements) and reactive (compensatory stepping corrections in response to external stimuli) components. Participant A’s MoCA score suggested a mild cognitive impairment and a worse quality of life than participant B, as per the PDQ-39.

### 3.2. Adherence and Adverse Events

The adherence to the supervised sessions was 100% (16 out of 16 planned sessions) for both participants over the eight-week period. The average duration of the session was 45 min (range 36–57 min) for participant A and 24 min (range 17–29) for participant B. No adverse events were reported during the sessions. However, participant A had two falls over the training period that were not related to the intervention (once in her room and once while walking outside). Participant B had one fall during the last week of training not related to the intervention (on the outside steps of the house).

### 3.3. Feasibility

Some technical issues were encountered during the supervised intervention. For participant A, two appointments were needed at the beginning of the intervention because the participant had difficulty understanding the procedure the first time. Another two days were interrupted during the intervention because the bandwidth was not available (in use by her husband for work purposes). For participant B, two sessions were re-scheduled due to a decrease in the bandwidth on the clinician’s site.

### 3.4. Acceptability

Acceptability to the intervention was measured through the Telemedicine Satisfaction Questionnaire (TSQ) that includes three factors or indicators of the performance of a healthcare service provided via telemedicine. Participant satisfaction with the intervention was considered very high as both participants scored the items at their highest levels, except for one item for each participant, where they simply agreed (4), rather than strongly agreed (5), with the given statements: “I do not need assistance while using the system” and “I obtain better access to health-care services by use of telemedicine” (both pertaining to factor 1 subgroup, i.e., quality of care provided). Both participants scored both factors 2 (similarity to face-to-face encounter) and 3 (perception of the interaction) at the maximum level.

### 3.5. Potential Efficacy

Table 2 shows the pre–post outcomes evaluated in this study. Overall, the two participants demonstrated an important carry-over adaptation in gait speed, balance, and perceived health-related quality of life. No change has been observed in aerobic capacity, as per the distance walked over 2 min.

As demonstrated in Table 2, participant A and B presented a meaningful improvement in self-selected gait speed (+0.72 and +0.19, respectively) and fast-paced gait speed (+0.65 and +0.28, respectively), which are higher than the previously demonstrated MDC in people with parkinsonism in both conditions (0.18 m/s for comfortable and 0.25 m/s for fast-paced gait speed) [52]. Participant A, with the worst baseline performance, improved her gait speed by two to three times that of her counterpart. Participant A also presented an improvement in the Mini-Best score (+7) much higher than participant B (+3) and higher than the MDC for older people with balance disorders and a history of falls, which normally ranges between 3.5 to 4 points [53,54].

No gain was observed for aerobic capacity measured by the two min walk test, which has an estimated MDC of 14.5 m in Parkinson’s Disease [50] and reported MDCs of around 10 m in similar populations (9.1 m in patients with dementia or Alzheimer’s Disease [55] and 19.2 m in Multiple Sclerosis [56]).

Regarding the perceived health status impacting quality of life, participants A and B scored better in the PDQ-39 questionnaire post-intervention than pre-intervention, with a decrease of 8 and 14 points in this scale, respectively (the lowest scores indicating a better quality of life). Item sub-scores in Table 2 suggest that the total score change is mainly accounted for by different dimensions for each participant: for participant A, these included activities of daily living, emotional wellbeing, and social support (increasing by 3 to 8 points each), with activities of the daily living dimension score only being considered meaningful [57]; for B, mobility, emotional wellbeing (4 to 6 points each) and, to a lesser extent, social support, cognition, and communication (1 to 2 points each), with the mobility dimension score only being considered meaningful [57].

## 4. Discussion

Our findings demonstrated that the BDJ exercise program delivered via a telerehabilitation system was feasible for these two participants, as per its successful implementation without major technical issues, no adverse events reported, and high adherence to the intervention; acceptable based on the satisfaction scores rated by our participants; and potentially effective to improve important markers of walking performance: self-selected and fast-paced gait speed, and static and dynamic balance. Finally, the intervention improved the participant’s overall quality of life, more specifically with tasks related to activities of daily living and mobility.

The COVID-19 pandemic has rapidly catalyzed the reorganization of many health care services toward telemedicine and telerehabilitation services [21,58], many of which have already been proven to be beneficial and cost-effective [19]. Consequently, telerehabilitation programs in PD, such as the one we pilot-tested in this study, should be further explored and tested in larger trials in order to implement rehabilitation programs in their most effective way.

The BDJ form of Qigong exercise has an advantage over other modalities of training programs due to its positive balance between desirable characteristics for home-based physical interventions (cost-effective, simple, self-paced, safe, of low intensity and duration, and not requiring additional equipment or a large space) and potential beneficial effects [26,27,28,29,59]. Due to these characteristics, Qigong-based exercises have been investigated in patients with PD and have been proven promising to alleviate non-motor and motor-related symptoms and their associated functional outcomes [28]. Additionally, it appears to have a significant impact on reducing levels of anxiety and depression in people with physical or mental conditions [60]. The effects of these forms of interventions go beyond the body and its function by integrating the physical exercise itself and components of mindful concentration, attention, and breathing patterns, which are typically impaired in patients with PD. Qigong-based interventions, as well as other mind-body exercises including yoga [61], seem therefore to be more advantageous than other traditional interventions, such as resistance or aerobic training, because they consider a holistic approach to the body and its different dimensions.

Interestingly, studies in PD showed that a Tai Chi intervention [62] and BDJ interventions [26,27], in comparison to a routine exercise program, improved physical function outcomes and reduced the incidence of falls within a six-month period. In a telerehabilitation context, preliminary data on post-stroke patients as part of a larger trial [40], demonstrated that an eight-week Tai Chi program led to significant improvements in balance, gait, fear of falling, motor function, and strength and postural control, and patients were satisfied with the intervention. However, mind-body exercise programs, such as BDJ and Tai Chi programs delivered via telerehabilitation, remain to be explored.

Promising preliminary results were observed in balance. As per the measure used to assess balance, based on the cut-off identified for older adults (16 out of 28) [63] and PD (20 out of 32) [64], both patients in our study were initially at risk, or nearly at risk, of falling and both experienced a significant improvement following the intervention, especially the participant with the poorest balance at baseline. The Mini-Best test, in comparison to other balance assessment tools, has been shown to be the most accurate test in identifying older adults (AUC = 0.84) [63] and patients with PD (AUC = 0.86) at risk of falling, with a very good reliability in individuals with PD. A previous meta-analysis on the efficacy of Qigong-based therapy in patients with PD [29] corroborates our findings, by demonstrating an improvement in walking ability, motor symptoms, and balance in favor of Qigong exercise when compared to other exercise programs.

An important gain could also be observed in gait speed, which was, once again, even more important to the patient demonstrating the worst performance at baseline. Indeed, a cohort of older adults with PD [65] demonstrated an average self-selected gait speed of around 1.10 m/s, and an average fast-paced gait speed around 1.53 m/s, which put our participants above the average post-intervention. The authors have also demonstrated that both self-selected and fast gait speed were strong predictors of falls (six-month history of falls) with a cut-off of 0.98 m/s and 1.32 m/s, respectively, which indicates that our participants would be very unlikely to be at risk of falling after the intervention. Improvements observed in gait speed were comparable to Tai Chi [31,62] and BDJ [28,29] interventions. A recent systematic review in PD [39] has failed to detect an effect on gait speed following virtual reality rehabilitation trainings, in comparison to conventional or traditional rehabilitation trainings, although a significant effect has been observed on stride length, balance, and mobility. Finally, as in this pilot trial, the same systematic review has also shown the significant effect of virtual reality rehabilitation trainings for people with PD on quality of life (PDQ-39).

In terms of aerobic capacity or endurance, the participants in our study had a poorer performance at baseline (112 and 151 m) than the average performance previously reported in mild-to-moderate PD patients (166.1 m) [50], and similar or slightly lower than the performance observed in patients with Multiple Sclerosis (149 m) [56]. No meaningful improvement was observed pre–post intervention. One may argue that unless there has been an increase in the participants’ amount of spontaneous physical activity or voluntary engagement in outdoor walking activities because of their improvement in balance and gait speed, there is no reason to believe that the intervention itself would directly lead to an increase in aerobic capacity, since this type of intervention does not include any specific aerobic component. A systematic review on Tai Chi and its effect on aerobic capacity in the general population [66] as well as previous studies on Tai Chi and BDJ in PD have either observed a small improvement or failed to observe an effect on aerobic capacity, estimated by submaximal tests [27,30,31,50]. It would, therefore, be interesting to explore in the larger trial which factors, if any, contribute to an improvement in aerobic capacity as a result of this type of physical exercise, and the extent of this contribution. The addition of a measure that estimates the participants’ level of physical activity and daily energy expenditure throughout the intervention duration, such as accelerometers or wearable sensors, could contribute to a better understanding of these potential interactions and associations. Finally, measures of gait quality (heel strike angular velocity, gait regularity, stride length, cadence) could also help to gain a better sense of the underlying mechanisms by which this type of intervention improve gait speed, walking performance, and aerobic capacity. Indeed, a previous study including individuals with PD has shown that progressively higher angular velocities were associated with a better cadence while walking [67]. Interestingly, in order to meet the recommended level of physical activity (i.e., 150 min/week of moderate-intensity aerobic physical activity), a previous study has found that the intensity needed to achieve the recommended amount of walking corresponds to a sustained cadence of at least 100 steps/min [68]. Eventually, patients who are able not only to walk faster, but also better adjust the quality of their gait, would engage more frequently in walking activities as a mean to improve their health.

Limitations are inherent to the design of this study. Results are based on the data of two participants only but were intended to demonstrate whether the proposed intervention could lead to measurable changes, as well as to identify potential barriers and challenges with the implementation and acceptability of this modality of intervention that could be addressed prior to moving the larger trial forward. Proof-of-concept trials are not powered to detect efficacy (statistical differences) but do allow for a power analysis to be done to estimate the sample size needed for the future trial to confirm efficacy. Based on the following assumptions, the sample size for the future trial should be set at n = 31: gait speed (at comfortable pace) as the main outcome (continuous primary endpoint); single-arm study; norms for the Canadian population (0.95 ± 0.19 m/s) from the Canadian longitudinal study on aging (CLSA) [69]; probability of a type I error of 0.05; statistical power of 80%; and at least 10% expected increase in gait speed following the intervention.

Additionally, we have no data on the perceived satisfaction from the healthcare provider’s perspective and no control groups to compare with (conventional therapy delivered via telerehabilitation and face-to-face BDJ rehabilitation), which would allow us to better nuance whether the patients were satisfied with the telerehabilitation system itself or with the exercise program delivered. The same limitations apply to the absence of comparison with an asynchronous telerehabilitation approach, the offline “store-and forward method”. Although most telerehabilitation studies address outcomes of synchronous, real-time time rehabilitation, there is some evidence that asynchronous telemedicine could also be effective and should be investigated in a future trial in terms of similar outcomes (feasibility, adherence, satisfaction, and efficacy) in this population.

Finally, the applicability and potential efficacy of this program might be limited to patients with no cognitive impairment only, as patients with cognitive issues were not eligible.

## 5. Conclusions

The results of this proof-of-concept study in people with PD provide preliminary evidence that a home-based BDJ program delivered via a telerehabilitation system is feasible from an implementation, safety, and compliance point of view, and acceptable as per the patients’ level of satisfaction. Finally, the telerehabilitation program seems potentially effective to improve important determinants of walking capacity, suggesting a tendency toward an improvement in overall quality of life, mainly explained by improvements in the physical dimensions of the construct.

## Figures and Tables

**Table 1:** Characteristics of the participants at study entry.

	Participant	
ID (W = Woman/M = Man)	A (W)	B (M)
Age (years)	75	74
Years since PD diagnosis	12	17
Hoehn and Yahr Stage	3	3
Use of medications ^#^	3	8
Living environment and use of assistive device	Home, with partner, with assistive device for outdoor walking only	Home, with partner, without walking aid
History of falls (n past year)	14	3
History of falls (n past month)	1	0
Balance (Mini-Best Test, n/28) *	11	17
Anticipatory (n/6)	1	4
Reactive postural control (n/6)	0	2
Sensory orientation (n/6)	4	2
Dynamic gait (n/10)	6	9
Global cognition (MoCA, n/30) **	20	26
Quality of life (PDQ-39, n/100) ***	78	68

^#^ Levodopa, COMT inhibitors, MAO-B inhibitors, dopamine agonists, anticholinergics, vasopressors, and/or antidepressants (considered continuous-use medication only); * Mini-Best (Mini Balance Evaluation Systems Test) has 14 items (distributed over four categories), each of them scored from 0 to 2 (0 indicating the lowest and 2 the highest level of function) with a total score range between 0–28; ** MoCA (Montreal Cognitive Assessment) score ranges between 0–30 with >26 considered to be normal, 18–25 indicating mild cognitive impairment, 10–17 indicating moderate cognitive impairment, and <10 indicating severe cognitive impairment; *** PDQ-39 Parkinson’s Disease Questionnaire-39, scored 0−100 (100 = maximum level of problems), the higher score indicating the worst quality of life. Item sub-scores can range between 0 and 4 (0 = never; 1 = seldom; 2 = sometimes; 3 = often; and 4 = always or cannot do at all).

**Table 2:** Responses to the BDJ telerehabilitation program.

Participant	A		B	
Timepoints	Pre-Intervention	Post-Intervention	Pre-Intervention	Post-Intervention
Self-selected gait speed (m/s)	0.77	1.48	1.49	1.67
Fast-paced gait speed (m/s)	0.99	1.72	1.64	2.00
Aerobic capacity (two min walk distance, m)	112	151	112	146
Balance (Mini-Best total score, n/28) *	11	17	18	20
Anticipatory (n/6)	1	4	3	5
Reactive postural control (n/6)	0	2	4	2
Sensory orientation (n/6)	4	2	4	3
Dynamic gait (n/10)	6	9	7	10
Quality of life (PDQ-39 total score, n/100) ^#^	78	68	70	54
Mobility	25	16	30	10
Activities of Daily Living	10	15	2	15
Emotional Wellbeing	14	11	11	7
Stigma	9	9	9	9
Social Support	3	1	0	0
Cognition	8	7	8	6
Communication	5	5	5	3
Bodily Discomfort	4	4	5	4

m/s, meters per second; m, meters; * Mini-Best (Mini Balance Evaluation Systems Test) has 14 items (distributed over four categories), each of them scored from 0 to 2 (0 indicating the lowest and 2 the highest level of function) with a total score range between 0–28; ^#^ PDQ-39 Parkinson’s Disease Questionnaire-39, scored 0−100 (100 = maximum level of problems), the higher score indicating the worst quality of life. Item sub-scores can range between 0 and 4 (0 = never; 1 = seldom; 2 = sometimes; 3 = often; and 4 = always or cannot do at all).

## Data Availability

Data is contained within the article.
